# Proteomic Analysis of Differentially Expressed Proteins Involved in Peel Senescence in Harvested Mandarin Fruit

**DOI:** 10.3389/fpls.2016.00725

**Published:** 2016-05-31

**Authors:** Taotao Li, Jingying Zhang, Hong Zhu, Hongxia Qu, Shulin You, Xuewu Duan, Yueming Jiang

**Affiliations:** ^1^Key Laboratory of Plant Resources Conservation and Sustainable Utilization, South China Botanical Garden, Chinese Academy of SciencesGuangzhou, China; ^2^Guangdong Provincial Key Laboratory of Applied Botany, South China Botanical Garden, Chinese Academy of SciencesGuangzhou, China; ^3^College of Life Science, University of Chinese Academy of SciencesBeijing, China; ^4^Zhangzhou Xiangcheng District Agricultural BureauFujian, China

**Keywords:** mandarin, proteomics, peel, senescence, Ca^2+^ signaling

## Abstract

Mandarin (*Citrus reticulata*), a non-climacteric fruit, is an economically important fruit worldwide. The mechanism underlying senescence of non-climacteric fruit is poorly understood. In this study, a gel-based proteomic study followed by LC-ESI-MS/MS analysis was carried out to investigate the proteomic changes involved in peel senescence in harvested mandarin “Shatangju” fruit stored for 18 days. Over the course of the storage period, the fruit gradually senesced, accompanied by a decreased respiration rate and increased chlorophyll degradation and disruption of membrane integrity. Sixty-three proteins spots that showed significant differences in abundance were identified. The up-regulated proteins were mainly associated with cell wall degradation, lipid degradation, protein degradation, senescence-related transcription factors, and transcription-related proteins. In contrast, most proteins associated with ATP synthesis and scavenging of reactive oxygen species were significantly down-regulated during peel senescence. Three thioredoxin proteins and three Ca^2+^ signaling-related proteins were significantly up-regulated during peel senescence. It is suggested that mandarin peel senescence is associated with energy supply efficiency, decreased antioxidant capability, and increased protein and lipid degradation. In addition, activation of Ca^2+^ signaling and transcription factors might be involved in cell wall degradation and primary or secondary metabolism.

## Introduction

Fruit ripening is a complex and genetically programmed process, which involves a series of organoleptic, physiological, and biochemical changes, resulting in the development of edible quality (Prasanna et al., 2007). However, ripening-associated changes also increase the susceptibility to physical injury and decrease resistance to microbial infection (Giovannoni, 2001), which cause enormous economic losses in some fruit crops. Therefore, an improved understanding of fruit ripening attributes may help to develop strategies to improve the nutritional and sensorial quality and reduce postharvest losses of fruit.

On the basis of ripening attributes, fruit are classified as climacteric or non-climacteric. Ethylene synthesis is essential for climacteric fruit ripening (Lin et al., 2009). Two systems (system-1 and system-2) are involved in ethylene biosynthesis. System-1 is responsible for the basal level of ethylene production in all plant tissues, whereas system-2 functions during climacteric fruit ripening and petal senescence (Lelievre et al., 2007). The transition from system-1 to system-2 results in the sharp increase in climacteric fruit ethylene biosynthesis, which initiates the ripening-related changes by a signal transduction cascade (Alexander and Grierson, 2002). In addition, a large group of transcription factors (TFs), such as NAC, WRKY, C2H2-type zinc finger, AP2/EREBP, and MYB, play important roles in fruit ripening and senescence (Lim et al., 2007; Asif et al., 2014). For non-climacteric fruit, no marked changes in respiration are observed and ethylene biosynthesis remains at very low levels (Alexander and Grierson, 2002). Some studies have linked abscisic acid to the regulation of non-climacteric fruit ripening and senescence (Ji et al., 2012; Wu J. et al., 2014). However, the ripening or senescence of non-climacteric fruit is still inadequately understood.

Proteomics provides an important approach to reveal the complexity of the post-harvest physiology of fruits at the protein level. Recently, a proteomics approach has been widely used to explore the molecular mechanism involved in ripening or senescence of fruits, including tomato (Wang et al., 2014), apple (Zheng et al., 2013), peach (Jiang et al., 2014), banana (Toledo et al., 2012), papaya (Huerta-Ocampo et al., 2012), mango (Wu H. X. et al., 2014), strawberry (Song et al., 2015), and bell pepper (Aizat et al., 2013). The elucidation of important physiological regulatory networks by such studies will allow optimization of storage and processing conditions to minimize product losses. In addition, the information obtained from proteomic studies will help to support the breeding and selection of novel cultivars to prolong shelf-life and improve nutrition and flavor quality (Pedreschi et al., 2013).

Citrus is one of the most economically important edible fruits worldwide. Citrus fruit are non-climacteric and can be classified into two classes, i.e., tight-skin citrus (such as sweet orange and pummelo) and loose-skin citrus (mainly mandarin) (Ding et al., 2015). In contrast to climacteric fruit, citrus fruit gradually senesce after harvest with dysfunction or malfunction of physiological processes in the fruit tissues, which finally leads to loss of storability (Ding et al., 2015). The recent publication of draft genomes for mandarin and sweet orange makes it possible to explore the mechanisms involved in citrus fruit senescence at the molecular level (Xu et al., 2013; Wu G.A. et al., 2014;). Several studies have analyzed the proteome of citrus fruit. Ma et al. (2013) reported that 2,4-D retarded Olinda Valencia orange senescence in postharvest citrus fruit by regulating the expression of stress-responsive and calcium-binding EF proteins. Yun et al. (2013) showed that the up-regulation of chloroplast stromal ascorbate peroxidase, ATP synthase beta subunit, and cytoplasmic malate dehydrogenase might be involved in heat treatment-induced disease resistance of citrus fruit. However, the ripening and senescence characteristics differed markedly among different cultivars. Therefore, it will be necessary to perform proteomic analyses of diverse citrus cultivars to unravel the ripening and senescence mechanism of citrus fruit.

To get more information on the senescence of harvested citrus fruit, the proteome of “Shatangju” mandarin peel at four stages of senescence were compared using a gel-based proteomic study (two-dimensional gel electrophoresis; 2-DE). The proteins that showed significant differences in abundance were sequenced using coupled mass spectrometry (liquid chromatography-electrospray ionization-tandem mass spectrometry; LC-ESI-MS/MS). In addition, selected postharvest physiological attributes were evaluated. The newly identified proteins enhance our understanding of the molecular basis of senescence of harvested non-climacteric fruit.

## Materials and methods

### Plant material

Mandarin (*Citrus reticulata* Blanco “Shatangju”) fruit were harvested at approximately 240 days after blooming from a commercial orchard in Zhaoqing, Guangdong Province, China. Fruit were selected for uniformity of shape, color, and size, with a total soluble solid of approximately 11.0%. The selected fruit were dipped for 3 min in 0.1% Sportak® (a.i. prochloraz; Bayer) fungicide solution to control postharvest diseases and allowed to air dry. The fruit were placed in polyethylene bags (0.03-mm film thickness) and stored at 25°C (80–85% relative humidity) for 18 days. Physiological parameters were evaluated and peel samples were taken at initiation of the experiment (day 0) and at three successive 6-day intervals during storage. The samples were immediately frozen in liquid nitrogen and stored at −80°C for protein extraction and analysis. Three independent biological replicates were conducted.

### Measurement of physiological parameters

Eight fruit were sealed inside a 4.2-L plastic container for 2 h at 25°C. Aliquots (1 mL) of headspace gas were withdrawn from each container and injected into a gas chromatograph (GC-9A; Shimadzu, Kyoto, Japan). Carbon dioxide (CO_2_) concentrations were determined using a thermal conductivity detector and a Poropak N column (Shimadzu). Ethylene concentrations were measured using a flame ionization detector and an HP-PLOT Q capillary column (Agilent Technologies, Palo Alto, CA, USA). Rates of ethylene production and respiration were expressed on a fresh weight basis.

Fruit color was measured with a Konica Minolta CR-400 colorimeter (Konica Minolta Co. Ltd., Tokyo, Japan) in the CIE-L^*^a^*^b mode. Color changes were quantified as the hue angle with the formula *h* = 180° + tan^−1^ (b^*^/a^*^) in accordance with Li et al. (2012). The contents of chlorophyll and carotenoids were determined spectrophotometrically following the method of Lichtenthaler (1987).

Membrane permeability was expressed as relative electrolyte leakage and measured in accordance with the method of Sun et al. (2012). Malondialdehyde (MDA) content was measured following the method described by Huang et al. (2013).

### Protein extraction from mandarin peel

Five grams of mandarin peel tissue from each subgroup was used for protein extraction following the method of Li et al. (2015a). The concentration of total proteins was determined using the Bio-Rad Protein Assay kit (Bio-Rad, Hercules, CA, USA).

### Two-dimensional electrophoresis and image analysis

Two-dimensional electrophoresis (2-DE) was performed in accordance with the method of Li et al. (2015a) with 17-cm IPGstrips (pH 5–8; BioRad). At least three independent gels for each biological replicate were run. After staining with Coomassie blue, PDQuest 2-DE software (version 8.0; Bio-Rad) was used to analyze the images. In brief, automated and manual matching functions were used to obtain the highest gel matching. The data were normalized using the total quantity of valid spots on the corresponding gel to account for quantitative variations in intensity of protein spots between samples. The normalized intensity of spots on three independent biological-replicate 2-DE gels was averaged and subjected to Student's *t*-test analysis. The spots that showed more than two-fold differences between two samples were considered to show significant changes in abundance and were excised for protein identification.

### In-gel protein digestion and protein identification by LC-ESI-MS/MS

In-gel protein digestion was performed in accordance with the method of Li et al. (2015b). LC-ESI-MS/MS was analyzed based on Orbitrap. Briefly, following protein digestion, peptide samples were desalted using a Strata X column (Phenomenex), vacuum-dried and then resuspended in a 200 μL volume of buffer A (2% ACN, 0.1% FA). After centrifugation, the supernatant was recovered to obtain a peptide solution with a final concentration of approximately 0.5 μg/μL. 10 μL supernatant was loaded on a LC-20AD nanoHPLC (Shimadzu, Kyoto, Japan) by the autosampler onto a 2 cm C18 trap column. Then, the peptides were eluted onto a 10 cm analytical C18 column (inner diameter 75 μm) packed in-house. The samples were loaded at 15 μL/min for 4 min, then the 91 min gradient was run at 400 nL/min starting from 2 to 35% B (98%ACN, 0.1%FA), followed by 5 min linear gradient to 80%, and maintenance at 80% B for 8 min, and finally return to 2% in 2 min.

The peptides were subjected to nanoelectrospray ionization followed by tandem mass spectrometry (MS/MS) in a LTQ Orbitrap Velos (Thermo) coupled online to the HPLC. Intact peptides were detected in the Orbitrap at a resolution of 60,000. Peptides were selected for MS/MS using collision induced dissociation (CID) operating mode with a normalized collision energy setting of 35%. Ion fragments were detected in the LTQ. A data-dependent procedure that alternated between one MS scan followed by ten MS/MS scans was applied for the ten most abundant precursor ions above a threshold ion count of 5000 in the MS survey scan with a following Dynamic Exclusion settings: repeat counts, 2; repeat duration, 30 s; and exclusion duration, 120 s. The applied electrospray voltage was 1.5 kV. Automatic gain control (AGC) was used to prevent overfilling of the ion trap; 1 × 104 ions were accumulated in the ion trap to generate CID spectra. For MS scans, the m/z scan range was 350–2000 Da.

Proteins were identified by using Mascot search engine (Matrix Science, London, UK version 2.3.02) against JGI_Citrus_clementina genome database (30796 sequences). A mass tolerance of 20 ppm for intact peptide and a 0.1 Da for fragmented ions were permitted, with allowance for one missed cleavages in the trypsin digests. Glu ->pro-Glu (N-term Q), Oxidation (M), Deamidated (NQ) as the potential variable modifications, and Carbamidomethyl (C), as fixed modifications were applied. The charge states of peptides were set to +2 and +3. Specifically, an automatic decoy database search was performed in Mascot by choosing the decoy checkbox in which a random sequence of database is generated and tested for raw spectra as well as the real database.

To reduce the probability of false peptide identification, peptides with ion scores greater than the “identity” score were counted as identified. Each confident protein identification involved at least one unique peptide. All matched peptides are shown in Table S1.

### Protein–protein interaction analysis

Protein–protein interaction (PPI) analysis of differentially expressed proteins during mandarin peel senescence was conducted following the method described by Dong et al. (2015) with minor modifications. Briefly, all proteins were subjected to a BLAST search against *Arabidopsis thaliana* data lodged in the STRING database (version 10.0; http://string-db.org). The matching proteins with a confidence score of at least 0.400 were used for PPI analysis. The PPI network was constructed and displayed using Cytoscape (version 3.0.2) software.

### Statistical analysis

Experiments were conducted with three biological replicates. The significance of differences in physiological parameters was tested by analysis of variance using SPSS (version 13.0; SPSS Inc., Chicago, IL, USA). Least significant differences were calculated to compare significant effects at the 5% level. Principal component analysis (PCA) of differentially expressed proteins at different stages of peel senescence was conducted using the “vegan” package in R version 3.1.0.

## Results

### Physiological characteristics of harvested fruit during storage

**Figure 1:**
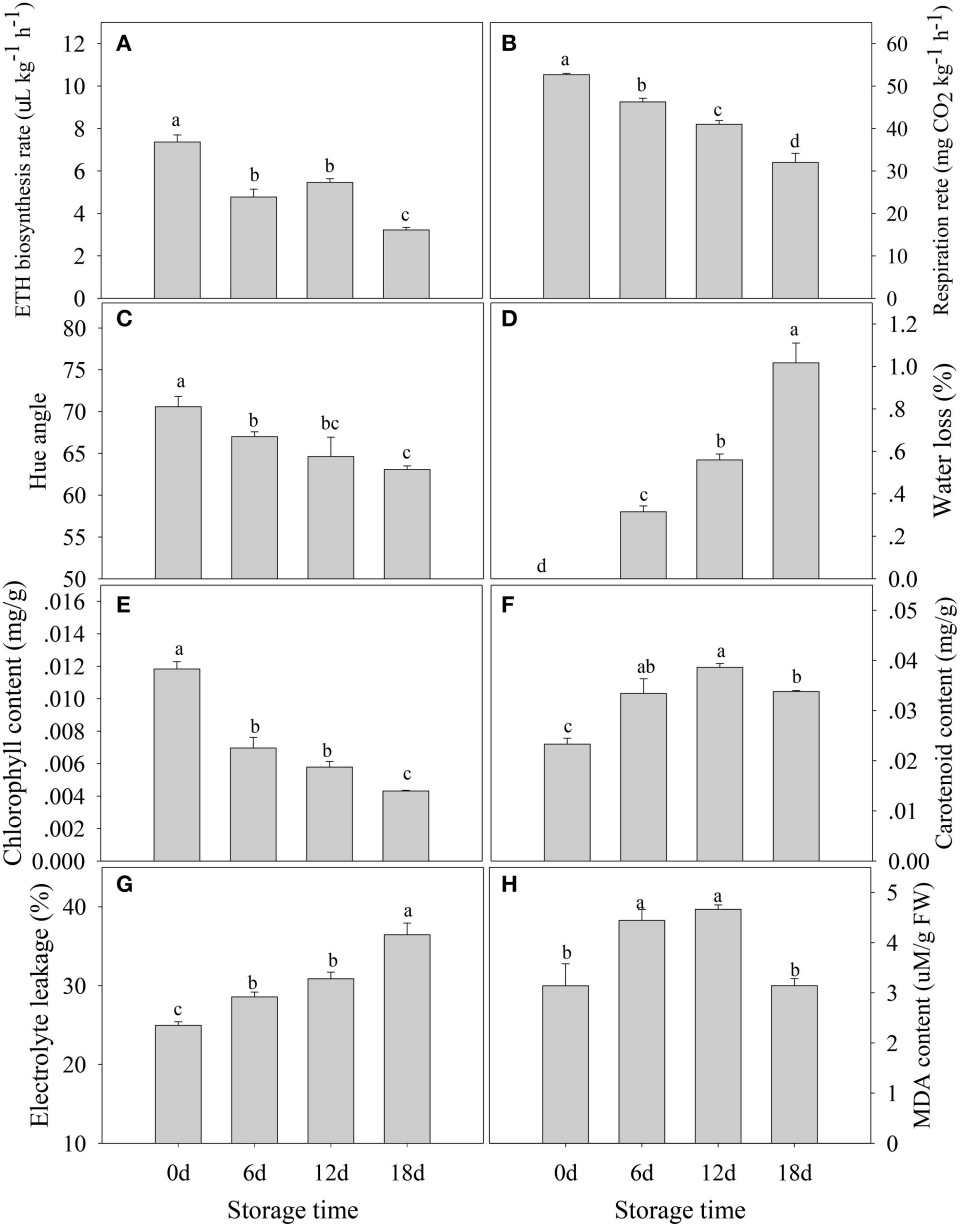
**Physiological characteristics of mandarin “Shatangju” fruit during storage. (A)** Ethylene biosynthesis rate; **(B)** respiration rate; **(C)** hue angle; **(D)** water loss; **(E)** chlorophyll content; **(F)** carotenoids content; **(G)** relative electrolyte leakage rate; **(H)** malondialdehyde (MDA) content. Ethylene biosynthesis rate, respiration rate, and water loss were measured for the whole fruit. Hue angle, chlorophyll content, relative electrolyte leakage rate, and MDA content were measured for the peel tissues only. Data presented are means ± standard errors (*n* = 3). Different letters above bars represent a significant difference (*p* < 0.05).

In contrast to climacteric fruit, the rates of ethylene production and respiration in harvested “Shatangju” mandarin fruit decreased with increasing duration of storage (Figures 1A,B). After storage for 18 days, the hue angle value (a measure of the change in peel color during senescence) decreased from the initial value of 70.58–63.07 (Figure 1C), and the percentage water loss increased significantly (Figure 1D). Consistent with the change in color, the chlorophyll content of the fruit peel decreased gradually during storage, accompanied by an increase in carotenoid content (Figures 1E,F). Relative electrolyte leakage is an index of cellular membrane integrity and fruit senescence. The initial (day 0) relative electrolyte leakage was 24.9%. After 18 days of storage, relative electrolyte leakage increased to 36.4% and MDA content showed a similar pattern (Figures 1G,H). Thus, “Shatangju” mandarin fruit exhibited typical non-climacteric senescence characteristics after harvest.

### Proteomic analysis of peel from harvested fruit during storage

**Figure 2:**
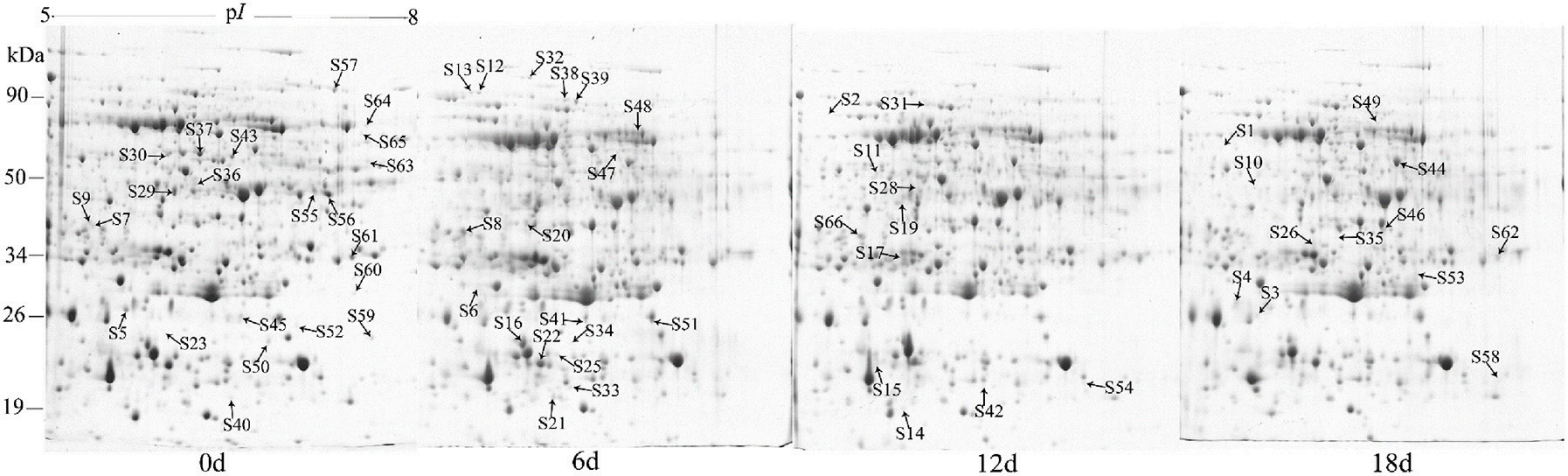
**Representative two-dimensional electrophoresis maps for the peel at different stages of senescence in mandarin “Shatangju” fruit**. The 2-DE maps show the total proteins isolated from the peel of harvested fruit after storage for 0, 6, 12, and 18 days. The labeled protein spots are those showing significant differences in abundance that were identified successfully.

**Figure 3:**
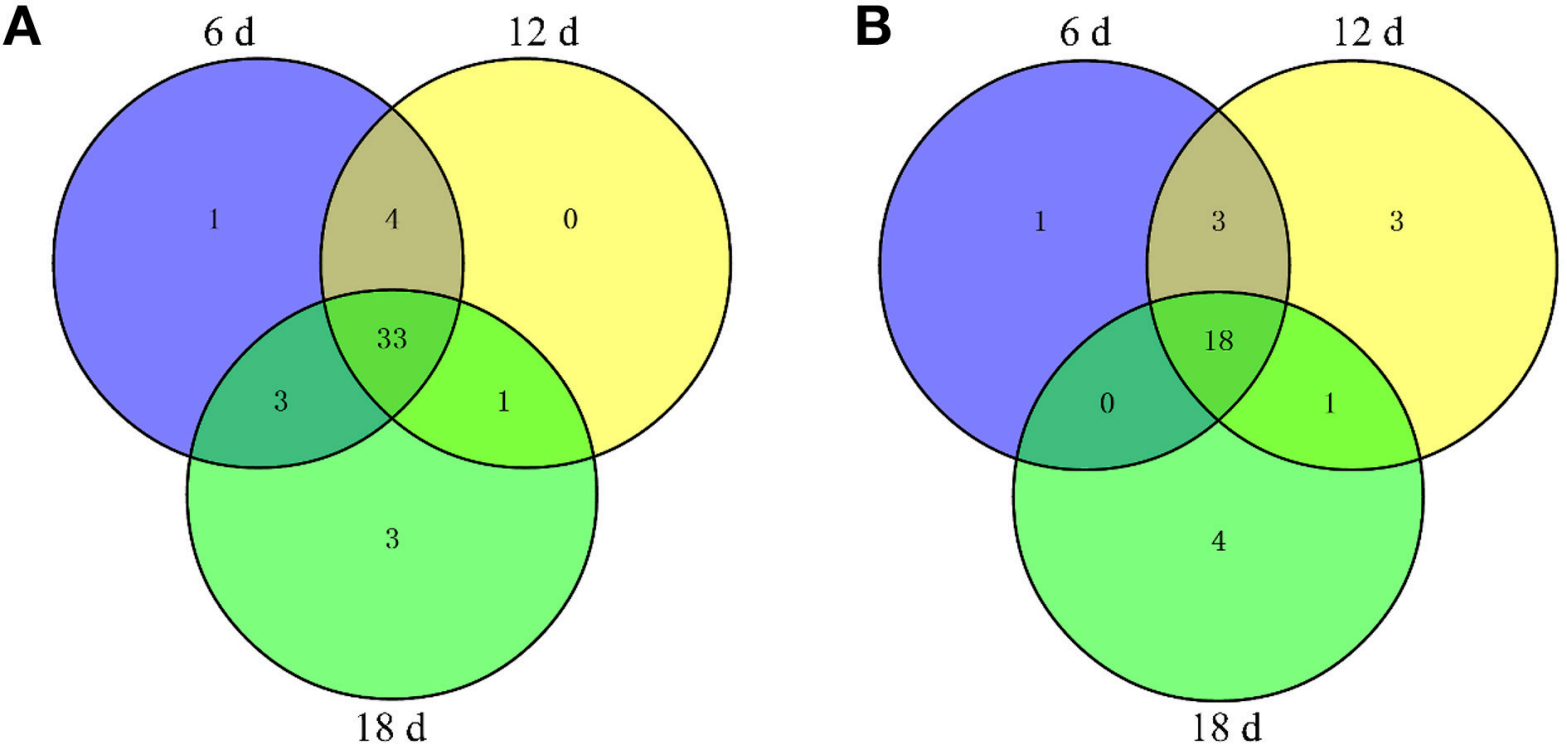
**Number of differentially expressed proteins in the peel at different stages of senescence in mandarin “Shatangju” fruit**. The number of **(A)** up-regulated proteins and **(B)** down-regulated proteins at different stages of senescence are indicated.

**Figure 4:**
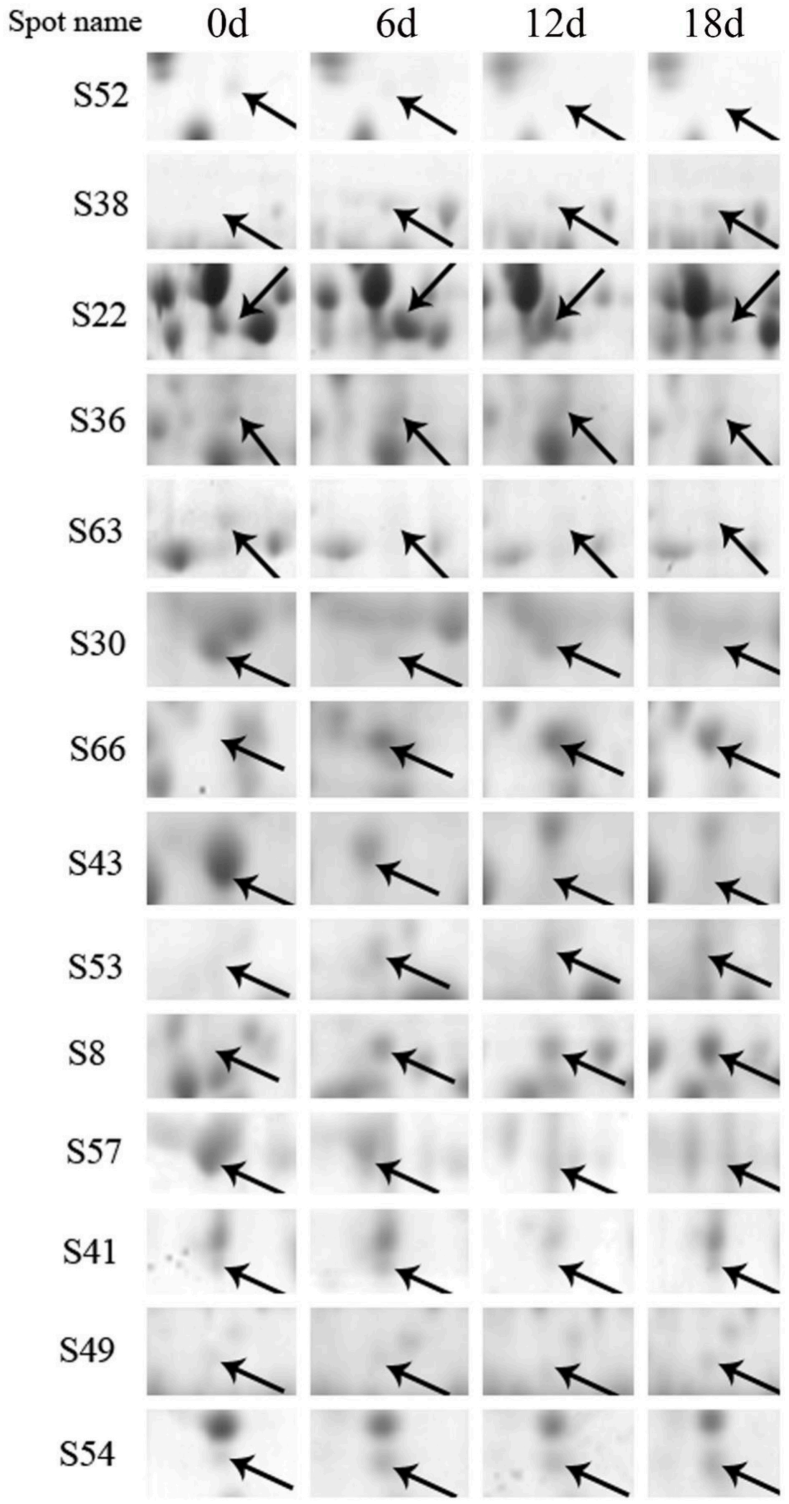
**High-magnification images of selected differentially expressed proteins**. Typical protein spots that showed significantly different abundances are indicated by arrows. Their sample names followed the manuscript annotation.

A 2-DE analysis was performed to investigate changes in the proteome of mandarin peel during senescence. Over 700 reproducible protein spots were detected by PDQuest 2-D analysis software (Figure 2). Representative 2-DE gels showing proteins isolated from the peel at different stages of senescence are shown in Figure S1. After normalization against the total density, 64 spots were differentially expressed (*P* < 0.05) with more than two-fold differences in abundance. Of these spots, 63 spots were identified successfully by LC-ESI-MS/MS analysis with ion scores greater than the “identity” scores for each peptide (Figure 2). Compared with the day 0 sample, 41 protein spots were up-regulated significantly and 22 were down-regulated in the day 6 sample. Thirty-eight protein spots were up-regulated significantly and 25 were down-regulated in the day 12 sample. After 18 days of storage, 40 protein spots were up-regulated significantly and 23 were down-regulated. Of the protein spots, 33 were up-regulated during the entire senescence process and 18 were down-regulated in all of the 6, 12, and 18 days samples (Figure 3). High-magnification views of some of the typical differentially expressed proteins are shown in Figure 4.

### Functional classification and expression patterns of identified proteins

**Figure 5:**
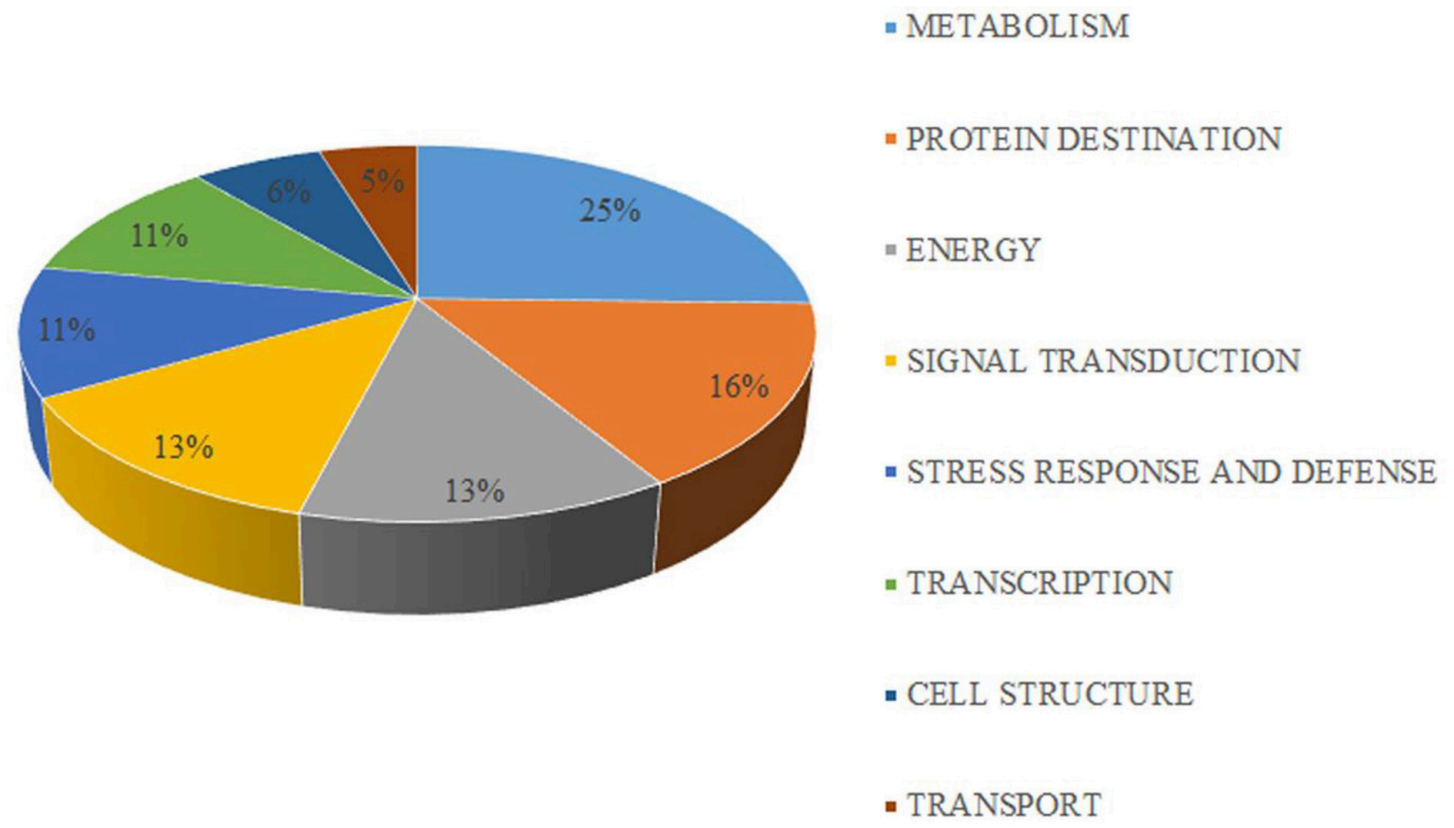
**Functional classification of differentially expressed proteins identified from mandarin “Shatangju” peel at different stages of senescence**. A total of 63 proteins were classified into biological process categories using the Blast2Go software.

The 63 differentially expressed proteins were classified into eight functional classes using Blast2Go software (Figure 5, Table 1). The major functional classes were metabolism, protein destination, energy, and signal transduction, which accounted for 25.4, 15.9, 12.7, and 12.7% of the differentially expressed proteins, respectively.

**Table 1:** **Identity of proteins differentially expressed during mandarin “Shatangju” peel senescence**.

**Sample name**	**Protein accumulation**	**MA**	**Protein ID**	**Protein description**	**Theo Mr/IP**	**2D Mr /IP**	**U-P/S**
**METABOLISM (16)**							
S1	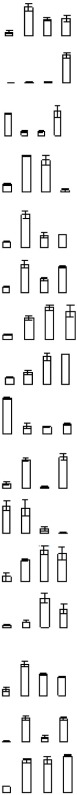	260	Ciclev10025791m	Phospholipase A 2A	43.26/4.97	69.9/5.17	2/2
S3		663	Ciclev10029169m	Lactoylglutathionelyase family protein/glyoxalase I family protein	27.33/8.64	28.69/5.51	11/82
S5		2242	Ciclev10002599m	Hypoxanthine-guanine phosphoribosyltransferase	20.68/5.64	30.02/5.58	8/12
S14		538	Ciclev10006131m	Lactoylglutathionelyase/glyoxalase I family protein	17.78/9.01	17.5/5.8	6/12
S39		1138	Ciclev10020504m	S-adenosylmethioninesynthetase 2	43.62/5.91	91.7/6.5	5/7
S41		576	Ciclev10000089m	Phospholipase D P2	128.65/6.77	28.15/6.55	1/1
S42		216	Ciclev10022058m	Nucleoside diphosphate kinase 2	25.71/8.99	19.75/6.64	5/17
S48		1511	Ciclev10019689m	Serine hydroxymethyltransferase 3	57.89/7.28	76.19/6.92	15/44
S56		4274	Ciclev10021104m	Hydroxyproline-rich glycoprotein family protein	37.37/6.95	53.26/7.36	5/43
S58		408	Ciclev10014585m	10-formyltetrahydrofolate synthetase	68.04/7.01	20.24/7.54	14/23
S60		281	Ciclev10014585m	10-formyltetrahydrofolate synthetase	68.04/7.01	32.32/7.54	4/5
S15		775	Ciclev10023414m	UDP-glucosyl transferase 73B1	55.89/7.14	22.49/5.64	1/1
S17		1484	Ciclev10029158m	S-adenosyl-L-methionine-dependent methyltransferases superfamily protein	27.84/5.37	38.66/5.8	5/6
S33		455	Ciclev10008765m	Thiazole biosynthetic enzyme, chloroplast (ARA6) (THI1) (THI4)	37.8/5.17	19.3/6.43	2/3
S34		410	Ciclev10030313m	UDP-glycosyltransferase 73B4	23.40/7.56	24.88/6.41	1/1
S54		721	Ciclev10031547m	Chorismate synthase, putative	48.3/8.22	20.08/7.3	1/1
**PROTEIN DESTINATION (10)**							
S4	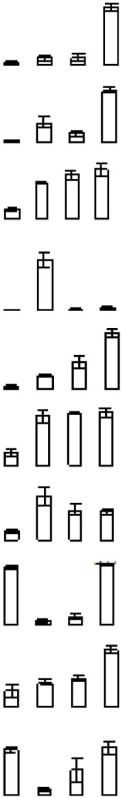	973	Ciclev10016547m	Heat shock protein 21	25.95/7.35	30.52/5.3	16/101
S10		284	Ciclev10020618m	Thioredoxin family protein	42.22/7.33	56.5/5.48	5/7
S11		541	Ciclev10020245m	ATP-dependent caseinolytic (Clp) protease/crotonase family protein	48.24/5.65	60.57/5.52	2/5
S13		285	Ciclev10031088m	Rotamase FKBP 1	64.07/4.92	95.28/5.31	25/94
S26		696	Ciclev10021693m	Rotamase CYP 4	29.31/8.66	41.99/6.12	3/10
S28		467	Ciclev10020618m	Thioredoxin family protein	42.22/7.33	56.66/5.95	10/12
S31		859	Ciclev10020618m	Thioredoxin family protein	42.22/7.33	88.65/6.09	6/13
S40		537	Ciclev10006181m	Methionine sulfoxide reductase B 2	15.33/6.74	18.78/6.68	3/5
S49		225	Ciclev10005062m	Ubiquitin-associated (UBA)/TS-N domain-containing protein	46.96/5.69	79.62/6.71	10/16
S50		1059	Ciclev10026390m	HSP20-like chaperones superfamily protein	26.78/9.33	24.86/6.93	6/12
**ENERGY (8)**							
S23	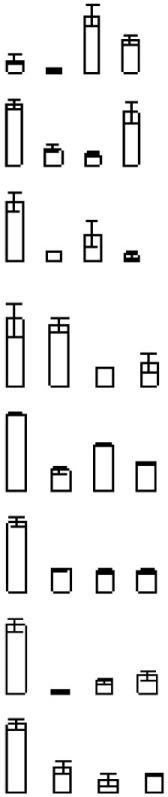	285	Ciclev10029115m	Triosephosphate isomerase	27.45/5.81	27.18/6.03	4/4
S29		1765	Ciclev10011726m	Enolase	48.07/5.52	53.23/6.1	8/12
S30		678	Ciclev10015332m	ATP synthase alpha/beta family protein	45.71/8.22	65/6.04	6/9
S36		853	Ciclev10020378m	Malate dehydrogenase	43.61/7.87	55.77/6.37	9/14
S37		3100	Ciclev10005101m	Phosphoglycerate kinase	42.36/6.2	64.86/6.44	12/16
S45		1343	Ciclev10002228m	Mog1/PsbP/DUF1795-like photosystem II reaction center PsbP family protein	27.72/6.53	28.91/6.74	1/1
S59		1242	Ciclev10032502m	Photosystem II subunit P-1	28.02/9.22	25.61/7.66	4/4
S63		569	Ciclev10028714m	Isocitrate dehydrogenase 1	39.57/8.44	63.14/7.63	3/3
**SIGNAL TRANSDUCTION (8)**							
S7	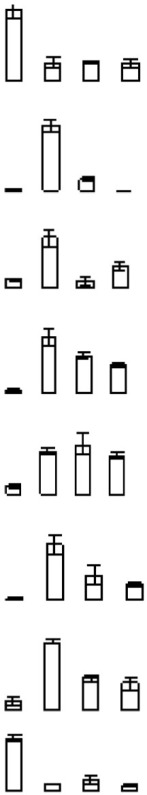	751	Ciclev10016618m	Plasma-membrane associated cation-binding protein 1	23.57/4.84	44.43/5.3	7/10
S12		457	Ciclev10009604m	Cyclin-dependent kinase-activating kinase assembly factor-related	20.78/4.64	95.43/5.4	1/1
S16		7087	Ciclev10030375m	Calcium-binding EF-hand family protein	25.96/8.43	25.21/5.83	1/2
S25		671	Ciclev10009604m	Cyclin-dependent kinase-activating kinase assembly factor-related	20.78/4.64	23.54/6.25	1/1
S35		165	Ciclev10029136m	Calcium-binding EF-hand family protein	28.12/6.36	43.37/6.38	3/3
S38		484	Ciclev10000174m	Leucine-rich repeat protein kinase family protein	106.69/6.78	92.41/6.35	1/1
S47		262	Ciclev10011848m	Calreticulin 3	49.27/6.45	68.91/6.78	17/26
S52		202	Ciclev10022440m	Regulatory component of ABA receptor 1	21.30/6.95	27.42/7.11	6/17
**STRESS RESPONSE AND DEFENSE (7)**							
S6	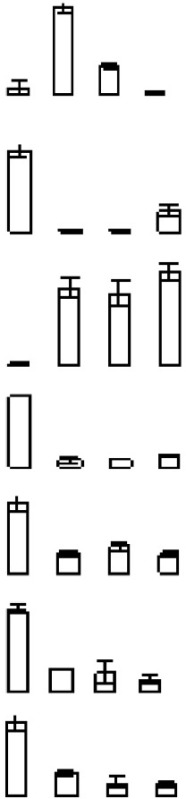	689	Ciclev10032324m	Iron/manganese superoxide dismutase family protein	31.96/7.71	33.55/5.36	3/3
S9		990	Ciclev10001194m	Thylakoidal ascorbate peroxidase	47.62/8.63	45.56/5.27	1/1
S19		176	Ciclev10026248m	Accelerated cell death 2 (ACD2)	31.32/5.82	50.92/5.78	12/24
S43		1294	Ciclev10011764m	Monodehydroascorbate reductase 1	47.22/6.21	62.39/6.68	4/4
S55		1966	Ciclev10000951m	Catalase 2	57.39/7.38	53.8/7.25	3/6
S61		2379	Ciclev10001685m	Ascorbate peroxidase 4	38.25/8.28	38.59/7.53	13/26
S64		417	Ciclev10000951m	Catalase 2	57.39/7.38	75.92/7.62	3/3
**TRANSCRIPTION (7)**							
S20	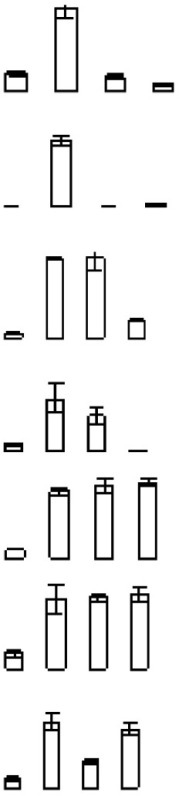	2115	Ciclev10021732m	RNA-binding (RRM/RBD/RNP motifs) family protein	28.39/5.26	49.23/5.91	9/36
S21		931	Ciclev10021732m	RNA-binding (RRM/RBD/RNP motifs) family protein	28.39/5.26	18.45/6.17	3/3
S22		8195	Ciclev10018462m	Transcription factor jumonji (jmjC) domain-containing protein	208.02/7.36	22.49/6	1/1
S32		253	Ciclev10016047m	bZIP protein	34.3/8.6	105/5.95	1/1
S44		5662	Ciclev10031606m	NAC domain containing protein 50	48.58/5.43	70.37/6.59	1/1
S46		718	Ciclev10028894m	NmrA-like negative transcriptional regulator family protein	34.22/6.64	45.23/6.74	1/1
S51		646	Ciclev10020365m	RNA-binding (RRM/RBD/RNP motifs) family protein	42.70/6.81	24.68/7.05	1/1
**CELL STRUCTURE (4)**							
S8	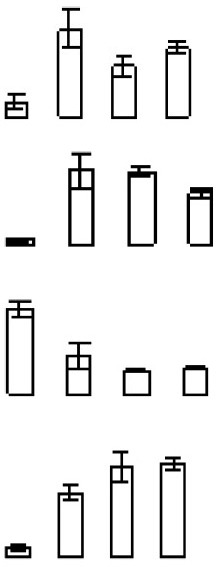	3089	Ciclev10025238m	Pectin methylesterase 3	63.96/9.25	45.07/5.21	1/1
S53		809	Ciclev10005561m	Xyloglucan endotransglucosylase/hydrolase 16	33.35/9.35	35.69/6.97	1/1
S57		1326	Ciclev10024598m	Plant invertase/pectin methylesterase inhibitor superfamily	43.01/6.9	89.42/7.43	1/1
S62		1468	Ciclev10013733m	Xyloglucan endotransglucosylase/hydrolase 24	29.61/7.75	39.46/7.53	1/1
**TRANSPORT (3)**							
S2	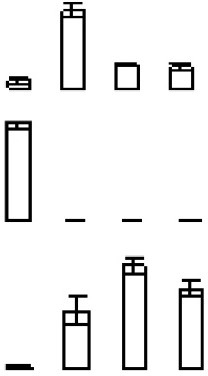	669	Ciclev10014785m	ATP binding cassette protein 1	62.44/5.58	84/5.15	6/7
S65		169	Ciclev10028483m	AAA-type ATPase family protein	48.60/7.59	73.06/7.6	9/13
S66		1621	Ciclev10033595m	ADP/ATP carrier 2	42.04/10.16	43.98/5.36	3/3

Protein accumulation is represented by the column configuration, and accumulation at 0, 6, 12, and 18 days is shown from left to right. MA, Maximum accumulation; Theo Mr/IP, Theoretical molecular mass and isoelectric point based on the amino acid sequence of the identified proteins; 2D Mr/IP, Experimental molecular mass and isoelectric point estimated from 2-DE gels; U-P/S, Unique peptide/spectrum. Protein information was obtained from the Citrus Genome Database (https://www.citrusgenomedb.org/species/clementina/genome1.0).

#### Metabolism

Eleven proteins (S1, S3, S5, S14, S39, S41, S42, S48, S56, S58, and S60) classified in the metabolism category are involved in primary metabolic processes (Table 1). Of these proteins, phospholipase A 2A (S1), lactoylglutathionelyase family protein/glyoxalase I family protein (S3), *S*-adenosylmethionine synthetase 2 (S39), phospholipase D P2 (S41), nucleoside diphosphate kinase 2 (S42), and serine hydroxymethyl transferase 3 (S48) were significantly up-regulated during the senescence of mandarin peel.

With regard to secondary metabolic processes, the five proteins identified (S15, S17, S33, S34, and S54) comprised two UDP-glucosyltransferases, one thiazole biosynthetic enzyme, one *S*-adenosyl-L-methionine-dependent methyltransferase superfamily protein, and one chorismate synthase. All of the proteins were up-regulated during peel senescence.

#### Protein destination

Ten proteins were involved in protein metabolic processes, of which three thioredoxins (TRXs; S10, S28, and S31), one rotamase CYP 4 (S26), one heat shock protein 21 (S4), and one ATP-dependent Clp protease (S11) were up-regulated after fruit harvest.

#### Energy

Of the eight proteins involved in energy metabolism, six proteins (ATP synthase alpha/beta family protein, malate dehydrogenase, phosphoglycerate kinase, Mog1/PsbP/DUF1795-like photosystem II reaction center PsbP family protein, photosystem II subunit P-1, and isocitrate dehydrogenase 1) were down-regulated, and one protein (triosephosphate isomerase) was up-regulated after fruit harvest.

#### Signal transduction

Eight proteins were classified in the signal transduction category. Interestingly, except for plasma-membrane associated cation-binding protein 1 (S7) and regulatory component of ABA receptor 1 (S52), all of the other proteins in this group, such as calreticulin 3 and calcium-binding EF-hand family protein, were significantly induced during fruit storage, especially early in the storage period.

#### Stress response and defense

In this category, six proteins involved in the scavenging of reactive oxygen species (ROS), comprising iron/manganese superoxide dismutase (S6), monodehydroascorbate reductase 1 (S43), ascorbate peroxidase (S9 and S61), and catalase 2 (S55 and S64), were down-regulated during senescence (Table 1). The accelerated cell death 2 (ACD2, S19) protein was significantly up-regulated.

#### Transcription

Seven transcription-associated proteins were identified. Of these proteins, three TFs, namely Jumonji C (JmjC) domain-containing protein (S22), NAC domain protein (S44), and NmrA-like negative transcriptional regulator (S46), were up-regulated (Table 1). In addition, three RNA-binding (RRM/RBD/RNP motifs) family proteins (S20, S21, and S51) were up-regulated early in the storage period.

#### Cell structure

Three cell wall modification-related proteins (pectin methylesterase 3, xyloglucan endotransglucosylase/hydrolase (XTH) 16 and XTH 24) were up-regulated during peel senescence. However, plant invertase/pectin methylesterase inhibitor (S57) was down-regulated.

#### Transport

Three proteins were classified in the transport category, consisting of ATP binding cassette protein 1 (S2), AAA-type ATPase family protein (S65), and ADP/ATP carrier 2 (S66).

### Principal component analysis (PCA) of differentially expressed proteins

**Figure 6:**
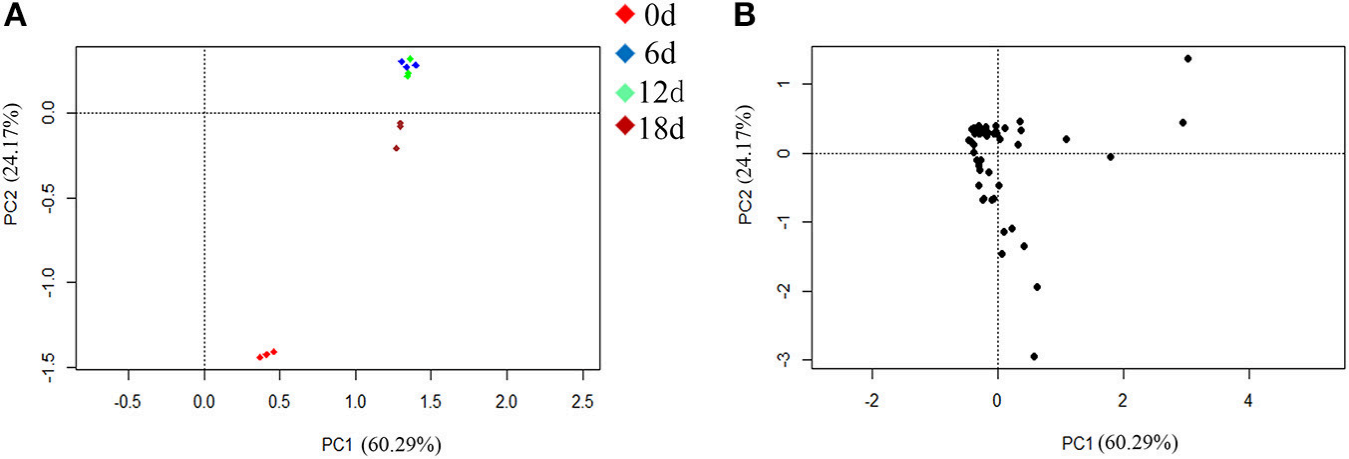
**PCA of differentially expressed proteins identified from peel of mandarin “Shatangju” fruit at different senescence stages. (A)** Score plot; **(B)** loading plot.

**Figure 7:**
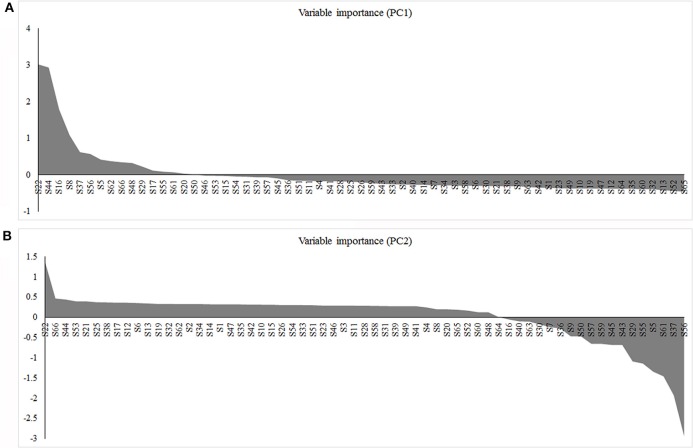
**Variable importance plots for the PCA of differentially expressed proteins. (A)** Loading scores for each identified protein on the first principal component axis (PC1). Proteins with a high positive PC1 loading score showed high abundance in samples from fruit stored for 6, 12, and 18 days. **(B)** Loading scores for each identified protein on the second principal component axis (PC2). Proteins with a high negative PC2 loading score showed high abundance in day 0 samples.

The PCA indicated that the different fruit storage durations could be discriminated, based on the differentially expressed proteins. The first principal component (PC1) explained 60.29% of the total variation and discriminated the biological samples on the basis of the storage duration, whereas PC2 explained 24.17% of the variation (Figure 6A). According to the score plot for PC1 and PC2, the 6 and 12 days samples could not be discriminated, whereas the 0 and 18 days samples were easily discriminated (Figure 6A). Overall, the PCA demonstrated senescence stage-specific differences in protein expression in the peel of stored mandarin fruit. To clarify which of the proteins contributed to the discrimination, variable importance plots were produced, based on the loading scores for PC1 and PC2 (Figure 6B). The variable importance plots indicated that only a small number of proteins contributed highly to the observed variability between the samples collected at different storage time-points (Figures 7A,B).

### Protein–protein interaction (PPI) network analysis of key differentially expressed proteins involved in mandarin peel senescence

**Figure 8:**
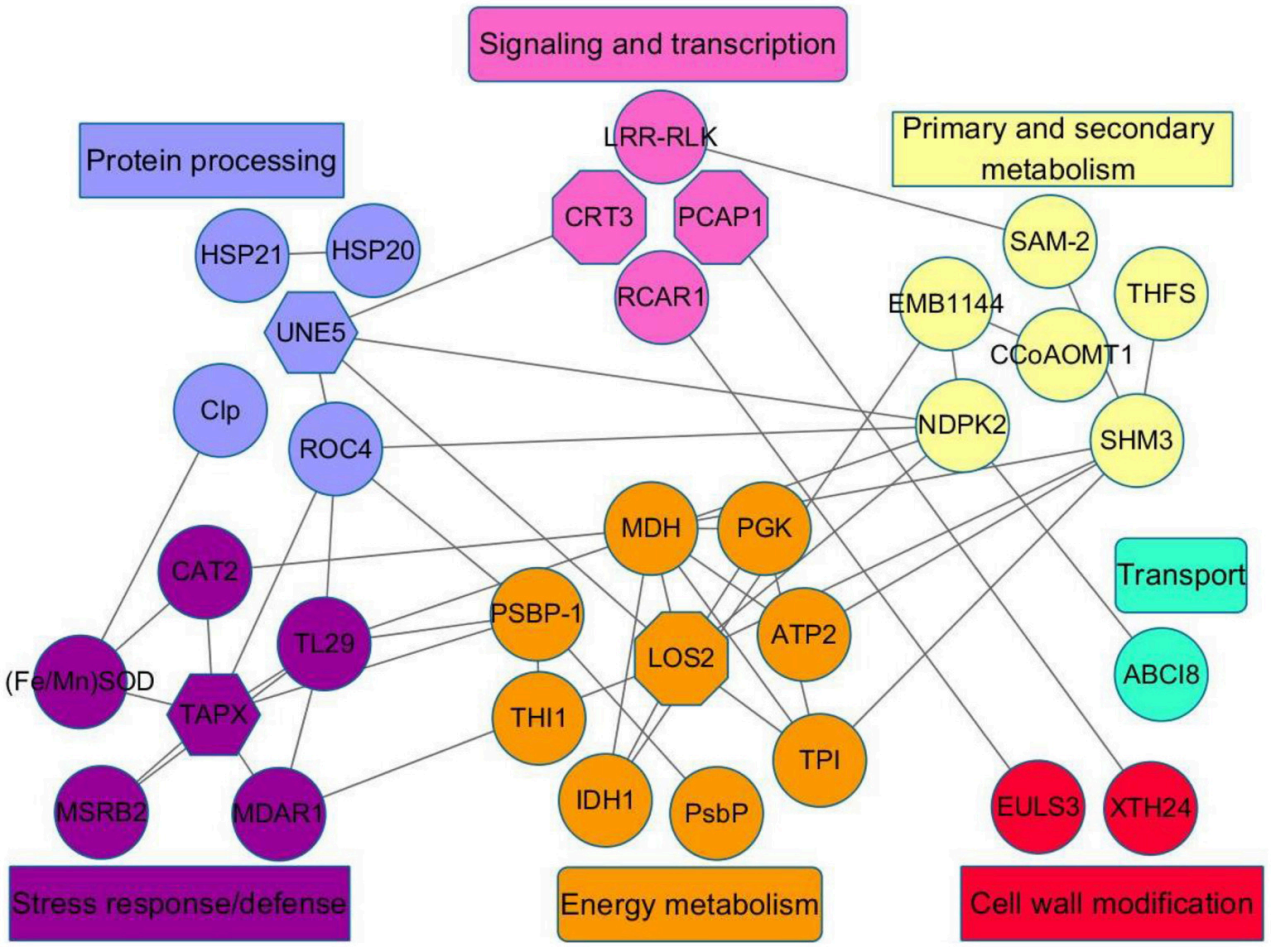
**Regulatory network of differentially expressed proteins identified from peel of mandarin “Shatangju” fruit at different stages of senescence**. Interactions among the differentially expressed proteins were analyzed using the STRING database with a confidence cutoff of 0.400. The interaction network was reconstructed using the Cytoscape software. The details of all nodes in the figure are listed in Table S2.

Mandarin peel senescence is a complex biological process that involves the interaction of numerous proteins. To further investigate the role of the differentially expressed proteins in the senescence process, we conducted a PPI analysis. The results indicated seven important functional categories with a total of 33 nodes (Table S2) mainly involved in signaling and transcription, protein processing, energy metabolism, stress response/defense, primary and secondary metabolism, cell wall modification, and transport (Figure 8). With the onset of peel senescence, the senescence signal was transduced by the Ca^2+^ signal pathway. Subsequently, protein processing became active, which might affect energy metabolism and response to stress. In addition, primary and secondary metabolism, and cell wall modification were also regulated by signaling and transcription pathways. Overall, the PPI analysis indicated that the differentially accumulated proteins involved in diverse physiological processes might interact and act synergistically during mandarin peel senescence.

## Discussion

Proteins differentially expressed in the peel tissues during mandarin fruit senescence were identified using a 2-DE approach. Comprehensive analysis of these proteins will help to elucidate the molecular mechanism involved in senescence of non-climacteric fruit.

### Proteins related to energy metabolism

Energy metabolism involves the release, transfer, storage, and use of energy in organisms. Glycolysis, the tricarboxylic acid (TCA) cycle, and oxidative phosphorylation are important energy-generating pathways that are active, at least in part, in almost all living organisms. In this study, four energy production-related enzymes showed significant differential expression in mandarin peel with increasing storage duration, namely phosphoglycerate kinase (PGK) (S37), enolase (ENO) (S29), isocitrate dehydrogenase (IDH) (S63), and malate dehydrogenase (MDH) (S36).

Phosphoglycerate kinase catalyzes the first ATP-generating step in the glycolytic pathway, whereas ENO catalyzes the last step of the glycolytic pathway, which generates phosphoenolpyruvate. We observed that PGK and ENO were significantly down-regulated during mandarin peel senescence, except for ENO at 18 days, which was inconsistent with the results reported for the ripening of some climacteric fruits. In these studies, increased carbon flux through glycolysis and increased glycolytic enzyme activities are reported at the transcript and protein levels (Borsani et al., 2009; D'Ambrosio et al., 2013). In a previous study we showed that PGK is involved in ethylene-induced chilling tolerance in harvested banana fruit (Li et al., 2015a).

Malate dehydrogenase reversibly catalyzes the conversion of malate to oxaloacetate in the citric acid cycle, whereas IDH catalyzes the oxidative decarboxylation of isocitrate in the TCA cycle. Both MDH and IDH are implicated in the generation of energy stored in the form of NADH in the TCA cycle. In this study, MDH and IDH were significantly down-regulated as mandarin peel senescence proceeded. Proteomic analyses suggest that up-regulated MDH or IDH are involved in ripening of the climacteric fruits tomato, apricot, and papaya (Rocco et al., 2006; Nogueira et al., 2012; D'Ambrosio et al., 2013). Thus, a discrepancy between climacteric fruit ripening and non-climacteric fruit senescence is suggested in terms of the TCA cycle.

In addition, one energy synthesis-related protein and one energy transport-related protein exhibited the opposite trend. ATP synthase alpha/beta subunits, an important component of the F1 ATP synthase complex, are vital for the final step of ATP synthesis from ADP and inorganic phosphate (Pedersen et al., 2000). The ADP/ATP carrier protein is a transport-related protein. The present results showed that one ATP synthase alpha/beta family protein (S30) was significantly down-regulated, whereas one ADP/ATP carrier protein (S66) was substantially up-regulated at the onset of senescence (Figure 4). These changes might be indicative of decreased ATP synthesis and increased energy consumption.

Energy supply is important for the maintenance of the homeostasis of proteins and membrane lipids (Liu et al., 2007; Sanchez-Bel et al., 2012). Mounting evidence suggests that senescence or physiological disorders of postharvest horticultural crops may be associated with inadequate energy supply and reduced efficiency of energy generation (Song et al., 2006; Wang H. et al., 2013; Li et al., 2016). Exogenous ATP treatment inhibits ROS accumulation, and maintains unsaturated fatty acid levels and membrane integrity, thus delaying senescence and deterioration of horticultural products (Yi et al., 2009, 2010). The down-regulation of PGK, MDH, IDH, and ATP synthase alpha/beta subunits inevitably would result in decreased efficiency of energy generation. Interestingly, both PGK (S37) and ADP/ATP carrier protein (S66) contributed highly to the variability in the proteome at different storage time-points, as indicated by the PCA (Figure 7). Therefore, it is suggested that reduced efficiency of energy generation and increased energy consumption led to energy depletion or ATP deficiency, which accelerated the senescence of mandarin peel.

### Proteins related to stress response and defense

Reactive oxygen species (ROS) are mainly generated by photosynthesis in chloroplasts, respiration in mitochondria, and other physiological processes involving electron transfer in plants (Tovar-Mendez et al., 2011). Most fruits after harvest undergo vigorous aerobic respiration, accompanied by accumulation of ROS. Previous research documented that fruit deterioration and senescence may be induced by imbalance between the production and scavenging of ROS (Macarisin et al., 2010). Damage caused by ROS includes protein oxidation, lipid oxidation, DNA strand breakages, and base modification, which all contribute to senescence. Malondialdehyde accumulation, a product of lipid peroxidation, and relative electrolyte leakage rate, which is an index of cellular membrane integrity, may reflect the extent of oxidative damage or senescence (Marangoni et al., 1996). The present results showed that the relative electrolyte leakage rate and MDA content significantly increased after fruit storage for 6 days (Figure 1), indicating that peel senescence had been initiated.

Redox regulation has been proposed as a response to oxidative stress during fruit ripening and senescence (Zheng et al., 2013). Antioxidant enzymes are important redox regulation systems and mainly consist of ascorbate peroxidase (APX), superoxide dismutase, catalase (CAT), glutathione peroxidase, and glutathione reductase (Moller, 2001). Ascorbate peroxidase is the main enzyme responsible for H_2_O_2_ removal in plant subcellular compartments. In this study, two APXs (S9 and S61) were identified and down-regulated significantly with the onset of peel senescence, which was inconsistent with results observed in tomato (Rocco et al., 2006), apricot (D'Ambrosio et al., 2013), and apple (Zheng et al., 2013). Catalase is also an important enzyme catalyzing the hydrolysis of H_2_O_2_. The abundance of two CATS (S55 and S64) decreased during the storage period, consistent with previous findings for the non-climacteric grape berries during ripening (Giribaldi et al., 2007). The down-regulation of APXs and CATs suggests that the capability to scavenge peroxide decreased in mandarin peel, which, in turn, accelerated senescence.

Monodehydroascorbate reductase (MDHAR) is an important component of the glutathione–ascorbatecycle, which is a crucial antioxidant system in plant cells to protect against damage induced by ROS. Li et al. (2010) reported that overexpression of chloroplastic MDHAR enhanced tolerance to methyl viologen-mediated oxidative stresses in tomato. Nahar et al. (2015) observed that exogenous spermidine alleviates low-temperature injury in mung bean seedlings by increasing the activities of glutathione-ascorbate cycle enzymes such as APX, MDHAR, dehydroascorbate reductase, and glutathione reductase. We observed that MDHAR (S43) was down-regulated during mandarin fruit storage (Figure 4), which consequently would influence the non-enzymatic components of the glutathione-ascorbate cycle.

The PCA indicated that S43 (MDHAR), S55 (CAT), and S61 (APX), which showed low abundances with increasing storage duration, contributed highly to discrimination between the different storage periods (Figure 7). Considering all of the above-mentioned results, we propose that down-regulation of these antioxidant enzymes played a role in peel senescence during fruit storage, which resulted in excessive accumulation of ROS and oxidative damage.

### Proteins related to signal transduction and transcription

Calcium, as a second messenger, plays an important role in plant growth, development and stress response. In the present study, three Ca^2+^ signaling-related proteins, consisting of two calcium-binding EF-hand family proteins (S16 and S35) and one calreticulin (S47), were differentially expressed during senescence. Calcium-binding EF-hand family proteins are Ca^2+^ sensors that recognize Ca^2+^signals. Calreticulin is a Ca^2+^-binding chaperone and modifies Ca^2+^ homeostasis in plants (Xiang et al., 2015). Overexpression of *CBP* gene decrease salt and osmotic tolerance in *Arabidopsis* (Chen et al., 2015). Therefore, we speculate that Ca^2+^ signaling initiates the senescence of harvested mandarin fruit.

Transcriptional regulation plays a vital role in fruit development and ripening as well as abiotic stresses. However, because of the limitations for protein identification and qualitative accuracy of 2-DE analysis, only a few differentially expressed TFs were detected in the current study. These TFs included a NAC domain-containing protein 50 (S44), JmjC domain-containing protein (S22), NmrA-like negative transcriptional regulator (S46), and bZIP (S32). NACs are plant-specific TFs that function in plant growth, development, and stress response (Zhao et al., 2010). In fruit, NACs are involved in the regulation of ripening via ethylene-signaling components (Shan et al., 2012) and low-temperature tolerance through ICE1-CBF (Shan et al., 2014). The present results showed that one NAC protein was substantially up-regulated as fruit senescence progressed. It is well known that non-climacteric ripening is ethylene independent. The involvement of NAC in mandarin fruit senescence possibly acts by regulating other genes rather than through ethylene signaling. The JmjC domain-containing protein shows histone demethylase activity (Tsukada et al., 2005). Histone modifications, such as methylation, acetylation, and phosphorylation, play an important role in epigenetic control of gene expression. Histone deacetylation is implicated in longan fruit senescence by interaction with ERF1 (Kuang et al., 2011). Up-regulation of the JmjC domain-containing protein may be beneficial for demethylation of certain TFs, which control the expression of senescence-related genes. The PCA also confirmed the important role of the JmjC domain-containing protein during mandarin peel senescence (Figure 7). Overall, the present proteomic data hinted that TFs may play important roles in regulating citrus peel senescence during fruit storage.

In addition, five transcription-related proteins, consisting of three RNA-binding proteins (RBPs) (S20, S21, and S51) and two cyclin-dependent kinase-activating kinase assembly factors (MAT1) (S12 and S25), were significantly up-regulated during mandarin fruit senescence. RBPs are associated with transcription, RNA processing, localization and stability, and translation (Shi et al., 2015). MAT1 mediates the assembly of cyclin-dependent kinase-activating kinase, which is functionally associated with the general TF IIH and is involved in transcription initiation and DNA repair. A role for RNA binding-proteins in the control of leaf senescence is implicated (Kim et al., 2008). There is little information available on the involvement of RBPs and MAT1 in fruit senescence. It is suggested that the up-regulated RBP and MAT1 proteins may be associated with the transcription or translation of certain senescence-responsive genes during mandarin peel senescence. The present PPI analysis also suggested these transcription-related proteins have important roles in citrus fruit senescence (Figure 8).

### Proteins related to protein metabolism

Fruit ripening involves protein modification, including biosynthesis, degradation, and folding. In this study, the differentially expressed proteins involved in protein metabolism mainly belonged to the TRX protein family, protease, and small heat shock protein. Moreover, these proteins may interact with other proteins to affect the mandarin peel senescence process (Figure 8).

Thioredoxins are small proteins with a conserved redox active-site WCG(P)PC, which regulate the redox status of target proteins (Holmgren et al., 2005). Thioredoxins play an important role in plant tolerance to oxidative stress by reducing the disulfide bond formed from the oxidation of cysteine residues by ROS (Dos Santos and Rey, 2006). It is well documented that oxidative stress may induce TRX expression (Shahpiri et al., 2009). We observed that three TRXs were significantly up-regulated in mandarin peel after 6 days of storage, indicating that the oxidation of protein cysteine residues occurred on a large scale. The increased expression of TRX proteins therefore would be beneficial for decreasing the extent of protein oxidation.

Caseinolytic protease (ClpP) is an energy-dependent serine protease, which unfolds protein substrates and transport them through a central pore and into the degradation chamber of ClpP. Up-regulation of ClpP is associated with ethylene-induced chilling tolerance in harvested banana fruit (Li et al., 2015a). An additional protein degradation-related protein, ubiquitin-associated (UBA)/TS-N domain-containing protein, was up-regulated in mandarin fruit, especially at the late storage stage. The ubiquitin–proteasome system is an important mechanism of protein degradation. Ubiquitin is covalently attached to a diverse array of target proteins and then degraded by proteasomes (Ramadan et al., 2015). Wang et al. (2014) reported that proteins involved in the ubiquitin–proteasome system were markedly induced during tomato fruit ripening. Given the significant up-regulation of ClpP (S41) and Ubac (S49) observed in the present study (Figure 4), it is suggested that ClpP and the UBA/TS-N domain-containing protein might be involved in protein degradation during the senescence of mandarin peel.

### Proteins related to cell wall degradation

The plant primary cell wall is a complex matrix of polysaccharides, mainly consisting of pectic and hemicellulosic polysaccharides and cellulose. Modification of the cell wall is important to attain commercial quality in some fruits (Brummell and Harpster, 2001). However, over-softening results in loss of marketability and decreased disease resistance, which in turn accelerates fruit senescence. Depolymerization of pectic and hemicellulosic polysaccharides plays an important role in fruit ripening, leading to disassembly of the cellulose-hemicellulose network and a decrease in fruit firmness (Wang D. et al., 2013). Xyloglucans are the main component of hemicellulosic polysaccharides. Thus, depolymerization of hemicelluosic polysaccharides is associated with the degradation of xyloglucans. The XTH family is believed to play a crucial role in this process (Miedes et al., 2013). Increasing evidence indicates that XTHs are involved in postharvest softening of many fruits (Han et al., 2015; Legay et al., 2015). In the present study, two XTH proteins (S53 and S62) were significantly up-regulated after 6 days of storage, which may have accelerated xyloglucan depolymerization and disassembly of the cellulose-hemicellulose network.

### Proteins related to primary metabolism

In senescence anabolic metabolism gives way to catabolic metabolism, resulting in aging and finally death of the tissue. In this study, a number of proteins associated with primary metabolite degradation were up-regulated, while a number of synthesis-related proteins were down-regulated.

Phospholipids are major components of all cell membranes. The degradation of phospholipids is mediated by a variety of phospholipases, including phospholipase A, C, and D. Phospholipase D cleaves phospholipids into phosphatidic acid and alcohol. Phospholipase D is reported to be involved in leaf senescence. Suppression of phospholipase D retards abscisic acid- and ethylene-promotedleaf senescence in *Arabidopsis* by attenuating lipid degradation (Jia et al., 2013; Jia and Li, 2015). Natalini et al. (2013) reported that cutting stimulated phospholipase D activities in tomato, in parallel with rapid softening and membrane degradation (as indicated by increased electrolyteleakage). In the current study, one phospholipase D and one phospholipase A2 were significantly up-regulated during mandarin fruit senescence, consistent with the implicated increase in membrane degradation (Table 1, Figures 1G,H). These results suggested that phospholipase may play an important role in mandarin peel senescence by degrading phospholipid in cell membranes.

Hydroxyproline-rich glycoproteins are important cell wall proteins, which form a continuous glycol network with non-cellulosic polysaccharides via covalent bonds or non-covalent interactions, thereby strongly contributing to cell wall architecture (Hijazi et al., 2014). The present results showed that one hydroxyproline-rich glycoprotein (S56) was dramatically down-regulated as mandarin peel senesced, which indicated that cell wall biosynthesis decreased. Considering the above-mentioned results and the up-regulation of cell-wall-degrading enzymes, the cell wall network was likely to be disrupted, which, in turn, accelerated peel senescence and resulted in loss of resistance to biotic or abiotic stresses.

### Overview of important biological processes involved in mandarin peel senescence

**Figure 9:**
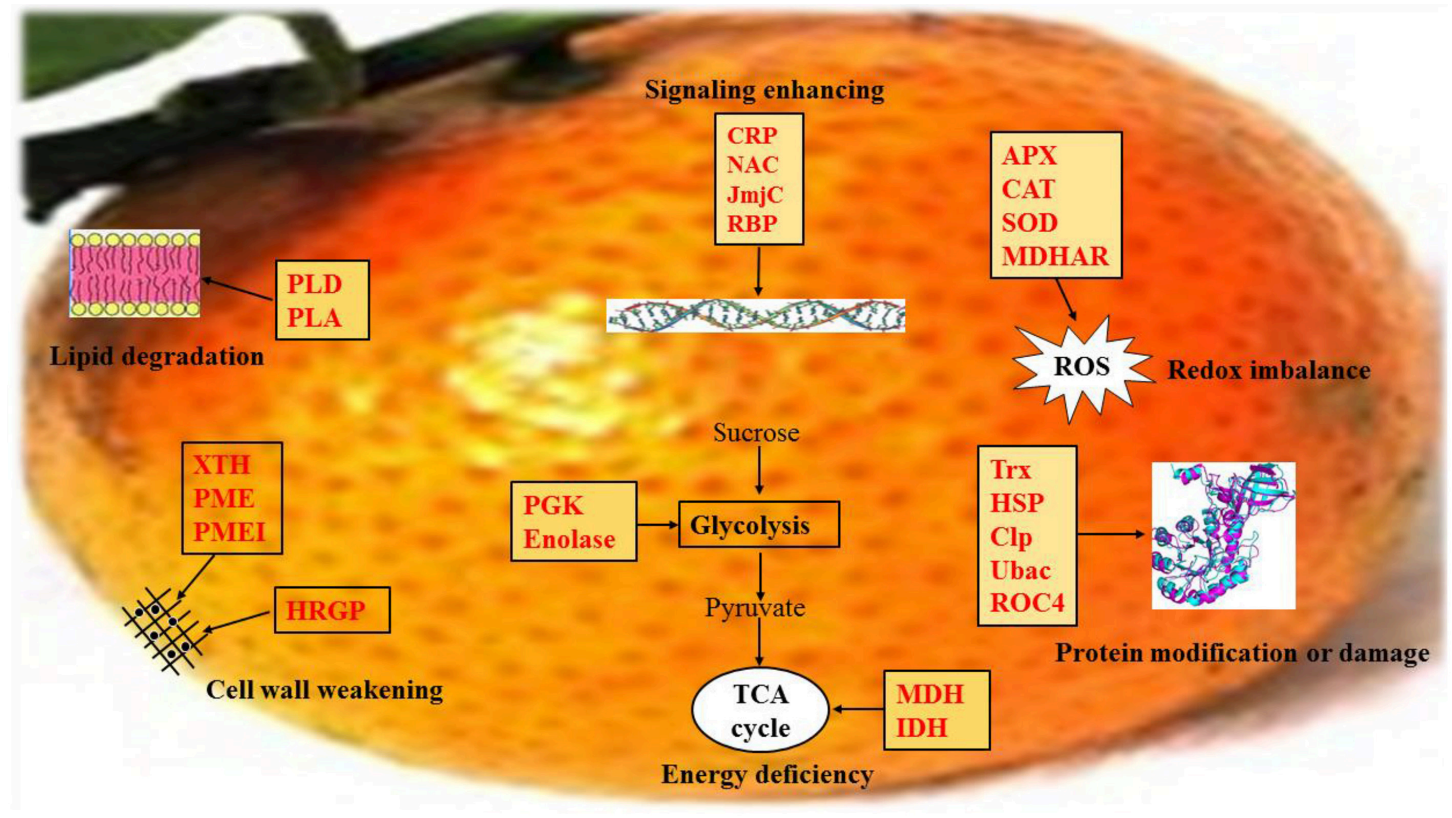
**Schematic of the important differentially expressed proteins involved in mandarin “Shatangju” peel senescence**. The proteins differentially expressed during peel senescence are indicated in red. CRP, Calcium related protein; NAC, *NAM, ATAF1/2, CUC1/2*; JmjC, Jumonji transcription factor; RBP, RNA-binding proteins; APX, Ascorbate peroxidase; CAT, Catalase; SOD, Superoxide dismutase; MDHAR, monodehydroascorbate reductase; PLD, Phospholipase D; PLA, Phospholipase A; XTH, Xyloglucan endotransglucosylase/hydrolase; PME, Pectin methylesterase; PMEI, Pectin methylesterase inhibitor; HRGP, Hydroxyproline-rich glycoprotein proteins; PGK, Phosphoglycerate kinase; Trx, Thioredoxins; HSP, Heat shock protein; Clp, Caseinolytic protease; Ubac, ubiquitin-associated (UBA)/TS-N domain-containing protein; ROC4, rotamase CYP 4; MDH, Malate dehydrogenase; IDH, Isocitrate dehydrogenase.

With consideration of the differentially expressed proteins identified in this study and previous reports, we propose a preliminary overview of important processes involved in mandarin peel senescence (Figure 9). After 6 days of storage, senescence had been initiated in the peel. With the onset of the senescence, the capability for ROS scavenging and the efficiency of energy generation was notably decreased, which resulted in accumulation of ROS and deficiency in energy supply, respectively. Excessive ROS accumulation causes oxidative damage to macromolecules, including proteins, lipids, and DNA. To cope with oxidative stress, proteins associated with protein modification were up-regulated to repair or degrade the damaged proteins. Some proteins involved in protein degradation or lipid repair require adequate energy supply; however, the deficiency in energy supply, in turn, accelerated protein damage and lipid degradation. During mandarin peel senescence, senescence-related signaling was likely activated by Ca^2+^signaling and TFs might be involved in the regulation of cell wall degradation, primary or secondary metabolism, and protein processing.

## Conclusion

In this study, proteins differentially expressed during mandarin fruit senescence were mainly associated with primary and secondary metabolism, signaling and transcription, protein modification, energy metabolism, and stress response and defense. The PPI analysis indicated that these proteins might contribute cooperatively to the senescence of the mandarin peel. It is suggested that Ca^2+^ signaling initiates senescence via transcription regulators. Energy deficiency and weakening of the antioxidant system accelerated senescence. Degradation of cell structural and functional components, including lipids, proteins, and cell wall polysaccharides, contributed directly to the progression of senescence. This study enhances our understanding of senescence in non-climacteric fruit and will contribute to the development of novel technologies to reduce postharvest losses of citrus fruit.

## Author contributions

TL and XD conceived and designed the study. TL, JZ, HZ, and SY performed the experiments and analyzed the data. TL and XD drafted the manuscript. All authors participated in the interpretation of data, the revision of the manuscript. All authors approved the submission and agreed to be accountable for all aspects of the work.

### Conflict of interest statement

The authors declare that the research was conducted in the absence of any commercial or financial relationships that could be construed as a potential conflict of interest.
