# Supplementary material for: Proteomic Analysis of Differentially Expressed Proteins Involved in Peel Senescence in Harvested Mandarin Fruit
Source: Front Plant Sci. 2016 May 31;7:725. doi: 10.3389/fpls.2016.00725 (PMC4885882; doi:10.3389/fpls.2016.00725)
Supplement: Supplementary file 2 [file Table2.DOCX]

| **Supplemental Table S2.** KOG annotation of DEPs from “shatangju” mandarin in the process of senescence. | | | |
| --- | --- | --- | --- |
| **Sample name** | **Protein descriptions** | **Node name in Figure** | **KOG description** |
|  | | | |
| S2 | ATP binding cassette protein 1 | ABCI8 | UPF0051 protein ABCI8 |
| S4 | heat shock protein 21 | HSP21 | heat shock protein 21 |
| S6 | Iron/manganese superoxide dismutase family protein | (Fe/Mn)SOD | Iron/manganese superoxide dismutase family protein |
| S7 | plasma-membrane associated cation-binding protein 1 | PCAP1 | plasma-membrane associated cation-binding protein 1 |
| S9 | thylakoidal ascorbate peroxidase | TAPX | L-ascorbate peroxidase |
| S10, S28, S31 | thioredoxin family protein | UNE5 | UNFERTILIZED EMBRYO SAC 5 |
| S12, S25 | cyclin-dependent kinase-activating kinase assembly factor-related | MAT1 | CDK-activating kinase assembly factor MAT1 |
| S17 | S-adenosyl-L-methionine-dependent methyltransferases superfamily protein | CCoAOMT1 | caffeoyl coenzyme A O-methyltransferase 1 |
| S23 | triosephosphate isomerase | TPI | triosephosphate isomerase |
| S26 | rotamase CYP 4 | ROC4 | rotamase CYP 4 |
| S29 | Enolase | LOS2 | LOW EXPRESSION OF OSMOTICALLY RESPONSIVE GENES 2 |
| S30 | ATP synthase alpha/beta family protein | ATP2 | ATP synthase subunit beta-3 |
| S33 | thiazole biosynthetic enzyme | THI1 | thiazole biosynthetic enzyme |
| S36 | malate dehydrogenase | MDH | malate dehydrogenase |
| S37 | phosphoglycerate kinase | PGK | phosphoglycerate kinase |
| S38 | Leucine-rich repeat protein kinase family protein | LRR-RLK | putative leucine-rich repeat transmembrane protein kinase |
| S39 | S-adenosylmethionine synthetase 2 | SAM-2 | S-adenosylmethionine synthase 2 |
| S40 | methionine sulfoxide reductase B 2 | MSRB2 | methionine sulfoxide reductase B 2 |
| S42 | nucleoside diphosphate kinase | NDPK2 | nucleoside diphosphate kinase 2 |
| S43 | monodehydroascorbate reductase 1 | MDAR1 | monodehydroascorbate reductase 1 |
| S45 | Mog1/PsbP/DUF1795-like photosystem II reaction center PsbP family protein | PsbP | PsbP domain-containing protein 4 |
| S47 | calreticulin 3 | CRT3 | calreticulin 3 |
| S48 | serine hydroxymethyltransferase 3 | SHM3 | serine hydroxymethyltransferase 3 |
| S50 | HSP20-like chaperones superfamily protein | HSP20 | HSP20-like chaperone |
| S52 | regulatory component of ABA receptor 1 | RCAR1 | abscisic acid receptor PYL9 |
| S54 | chorismate synthase | EMB1144 | chorismate synthase |
| S55,S64 | catalase 2 | CAT2 | catalase 2 |
| S56 | hydroxyproline-rich glycoprotein family protein | EULS3 | Euonymus lectin S3 |
| S58, S60 | 10-formyltetrahydrofolate synthetase | THFS | Formate--tetrahydrofolate ligase |
| S59 | photosystem II subunit P-1 | PSBP1 | photosystem II subunit P-1 |
| S61 | ascorbate peroxidase 4 | TL29 | thylakoid lumen 29 |
| S62 | xyloglucan endotransglucosylase/hydrolase 24 | XTH24 | xyloglucan:xyloglucosyl transferase |
| S63 | isocitrate dehydrogenase 1 | IDH1 | isocitrate dehydrogenase 1 |
