# Supplementary figures and images for: Proteomic Analysis of Differentially Expressed Proteins Involved in Peel Senescence in Harvested Mandarin Fruit
Source: Front Plant Sci. 2016 May 31;7:725. doi: 10.3389/fpls.2016.00725 (PMC4885882; doi:10.3389/fpls.2016.00725)

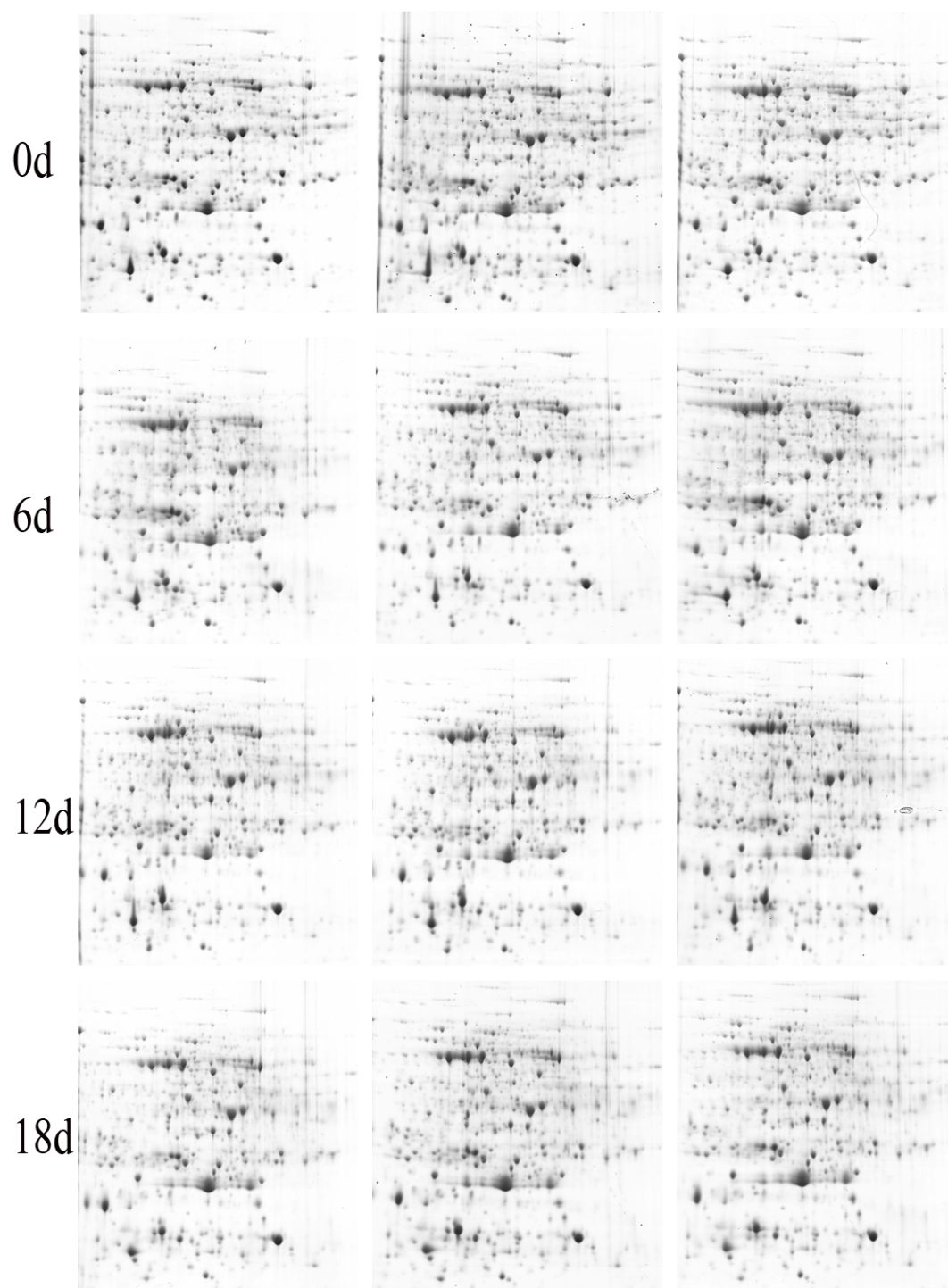

**FIGURE S1** The two-dimensional electrophoresis maps from different replicates.

Supplement: Supplementary file 3 [file Image1.PDF]
